# High myopia induced by form deprivation is associated with altered corneal biomechanical properties in chicks

**DOI:** 10.1371/journal.pone.0207189

**Published:** 2018-11-12

**Authors:** Byung Soo Kang, Li-Ke Wang, Yong-Ping Zheng, Jeremy A. Guggenheim, William K. Stell, Chea-su Kee

**Affiliations:** 1 School of Optometry, The Hong Kong Polytechnic University, Hong Kong SAR, China; 2 Department of Biomedical Engineering, The Hong Kong Polytechnic University, Hong Kong SAR, China; 3 School of Optometry & Vision Sciences, Cardiff University, Cardiff, Wales, United Kingdom; 4 Department of Cell Biology and Anatomy and Department of Surgery, Cumming School of Medicine, University of Calgary, Calgary, Alberta, Canada; Bascom Palmer Eye Institute, UNITED STATES

## Abstract

The cornea is a soft, transparent, composite organic tissue, which forms the anterior outer coat of the eyeball. Although high myopia is increasing in prevalence worldwide and is known to alter the structure and biomechanical properties of the sclera, remarkably little is known about its impact on the biomechanics of the cornea. We developed and validated a novel optical-coherence-tomography-indentation probe–to measure corneal biomechanical properties *in situ*, in chicks having experimentally-induced high myopia, while maintaining intraocular pressure at levels covering the physiological range. We found that the cornea of highly myopic chicks was more steeply curved and softer, at all tested intraocular pressures, than that in contralateral, non-myopic eyes, or in age-matched normal, untreated eyes. These results indicate that the biomechanical properties of the cornea are altered in chicks developing experimentally-induced myopia.

## Introduction

The cornea is a distensible, extracellular matrix-rich tissue that provides nearly 60% of the human eye’s focusing power. Anatomically, the cornea merges with the posterior coat of the eye, the sclera, and the two tissues share many structural properties. The cornea has long been a primary target for surgical intervention and refractive correction, and cumulative evidence using different approaches has indicated the importance of understanding corneal biomechanical properties in the diagnosis and management of intervention involving corneal tissue[1]. However, despite extensive studies on the role of corneal biomechanics–in the diagnosis[2, 3] and treatment[4] of keratoconus, in deriving accurate intraocular pressure (IOP) measurements[5], and in evaluating the outcomes of refractive surgeries[6, 7]–comparatively little is known regarding whether the biomechanical properties of the cornea are altered in the development of high myopia. This contrasts with the wealth of information that has been obtained about the structural and biomechanical changes occurring in the sclera of eyes developing high myopia, which include tissue loss[8], altered distribution of collagen fibers of varying diameters[8], collagen degradation[9, 10], and modification of biomechanical properties[11–13].

The precise shape of the cornea is governed by the biomechanical properties of its thick and regularly structured stromal layer. Little is known about whether the altered structure and ultrastructure of the sclera[8] in myopic eyes are accompanied by comparable changes in the cornea’s biomechanical properties. Nevertheless, there is ample evidence that the corneal *structure* is altered in myopic eyes. First, human myopia is associated with an increased corneal curvature and reduced thickness[14–18] (however, see also contradictory findings[19–21]). Second, in animal models, many experimental treatments–form deprivation (FD), optical defocus, constant lighting, spectral composition of the light source, and high illuminant lighting conditions–alter not only the eye’s axial dimensions, but also the anterior corneal shape[22–25]. These results indicate the involvement of the anterior segment during refractive-error development, and they highlight the importance of understanding whether changes in the biomechanical properties of cornea underlie its altered shape in myopic eyes.

In this study, we incorporated optical coherence tomography (OCT)[26, 27] into our indentation system[28], and we used this novel OCT-indentation system to determine the impact of FD-induced high myopia on *in-situ* corneal biomechanical properties (CB), while IOP was maintained constant at one of three values in the physiological range. This system was designed with attention to the anatomical features of our target animal model, the chicken; namely: 1) the indenter probe was miniaturized (1 mm diameter) because of the steep corneal curvature in our small animals; 2) crystalline lens surface was used as a reference (see details in Methods), to avoid confounding error due to eyeball movement during corneal indentation[29]; and 3) time-domain OCT (TD-OCT) was incorporated, to provide fast, high-resolution tracking of corneal and crystalline lens surfaces during indentation at different IOPs. We chose TD-OCT as our first approach, because its reference arm allows an extended depth of detection, and because it costs less than frequency-domain OCT (FD-OCT). Data collected from this system were used to calculate corneal tangent modulus, by integrating with corneal geometrical parameters (corneal thickness and curvature) measured with other instruments[28–30]. As shown in this paper, our novel system is sensitive enough to measure small but significant changes in corneal biomechanical properties of highly myopic chicks.

## Materials and methods

### Animals

Sixteen White Leghorn chicks (*Gallus gallus domesticus*) were obtained from the Centralized Animal Facility of The Hong Kong Polytechnic University. Three batches of 5–6 chicks each were raised in a cage (75 cmx45 cm) illuminated by fluorescent tubes (150 lux at chick’s eye level, 12h:12h light-dark cycle with lights on from 0700 to 1900) in a temperature-controlled (25°C) room. Food and water were provided *ad libitum*. All experiments were conducted in accordance with the ARVO Statement for the use of Animals in Ophthalmic and Vision Research, and the protocols were approved by the Animal Subject Experiment Subcommittee of the Hong Kong Polytechnic University (#14-15/28).

### Form-deprivation myopia (FDM)

To induce FDM, a Velcro ring was glued to the feathers around the right orbit of 12 chicks on post-hatching day 5 (P5), and matching Velcro rings with plastic-molded translucent diffusers (thickness = 0.5 mm, diameter = 12 mm, average light transmission = 30%) were attached. In the subsequent one-week treatment period, the diffusers were removed daily for cleansing. The left eyes served as untreated control eyes. Four age-matched chicks without any treatment served as an age-matched normal group.

### Ocular biometric measurements

Refractive status, corneal parameters, and ocular axial dimensions of chicks were measured at P12 by a modified Hartinger refractometer[23], a custom-made videokeratography system (VKS)[31], and a high-resolution A-scan ultrasonography system[32], respectively. The measurements always started with VKS at 07:00–08:00 when chicks were alert, followed by refractions and A-scan ultrasonography when chicks were anesthetized. The three measurements were completed by 11:00. The protocols for these methods have been described in details elsewhere[23, 31, 32], and a brief description of each method follows.

### Videokeratography system (VKS)

After the pupillary center was aligned (concentric) with the Placido rings, a consecutive series of 500–800 frames was captured via multiple-shot mode, using a CCD camera for image analysis. Four or more images per eye were selected manually for image processing, on the basis of objective criteria described elsewhere[31] (viz., a minimum of 15 sharply focused Placido rings, with maximal ring-to-ring width). Mean corneal curvatures (average of the two principal power meridians) were calculated from these images through a custom-written MATLAB algorithm and averaged using power vector analysis[33].

### Hartinger refractometer

Refractive status was measured along the pupillary axis, using a modified Hartinger refractometer[23], while chicks were anesthetized by isoflurane inhalation (1.5% in O_2_, with oxygen flow rate of 1.5 L/min). Three measurements per eye were made and averaged for the spherical equivalent, using power vector analysis[33].

### A-scan ultrasonography

Ocular axial dimensions were measured using an A-scan ultrasonographer (GE Panametrics, U.S.) integrated with a 50 MHz focused high-frequency polymer transducer (PVDF; PI50-2-R0.50; GE Panametrics, U.S.). A-scan ultrasonography has been verified as an effective tool for measuring the axial dimensions of chicks’ ocular components[32] and is widely used in this field. After the chick was anesthetized, a drop of artificial tear fluid (Lacryvisc; Alcon, France) was applied to the cornea, to minimize irritation by the ultrasound-interfacing gel (Aquasonic; Parker Laboratories, U.S.). Fifty data sets per eye were collected by a data-collection card, installed in a computer, at a sampling rate of 500 MHz. These data were later analyzed, using a custom-written algorithm to identify peaks representing the borders between the ocular components[32], and averaged.

### Optical-coherence-tomography-indentation probe system

**Fig 1:**
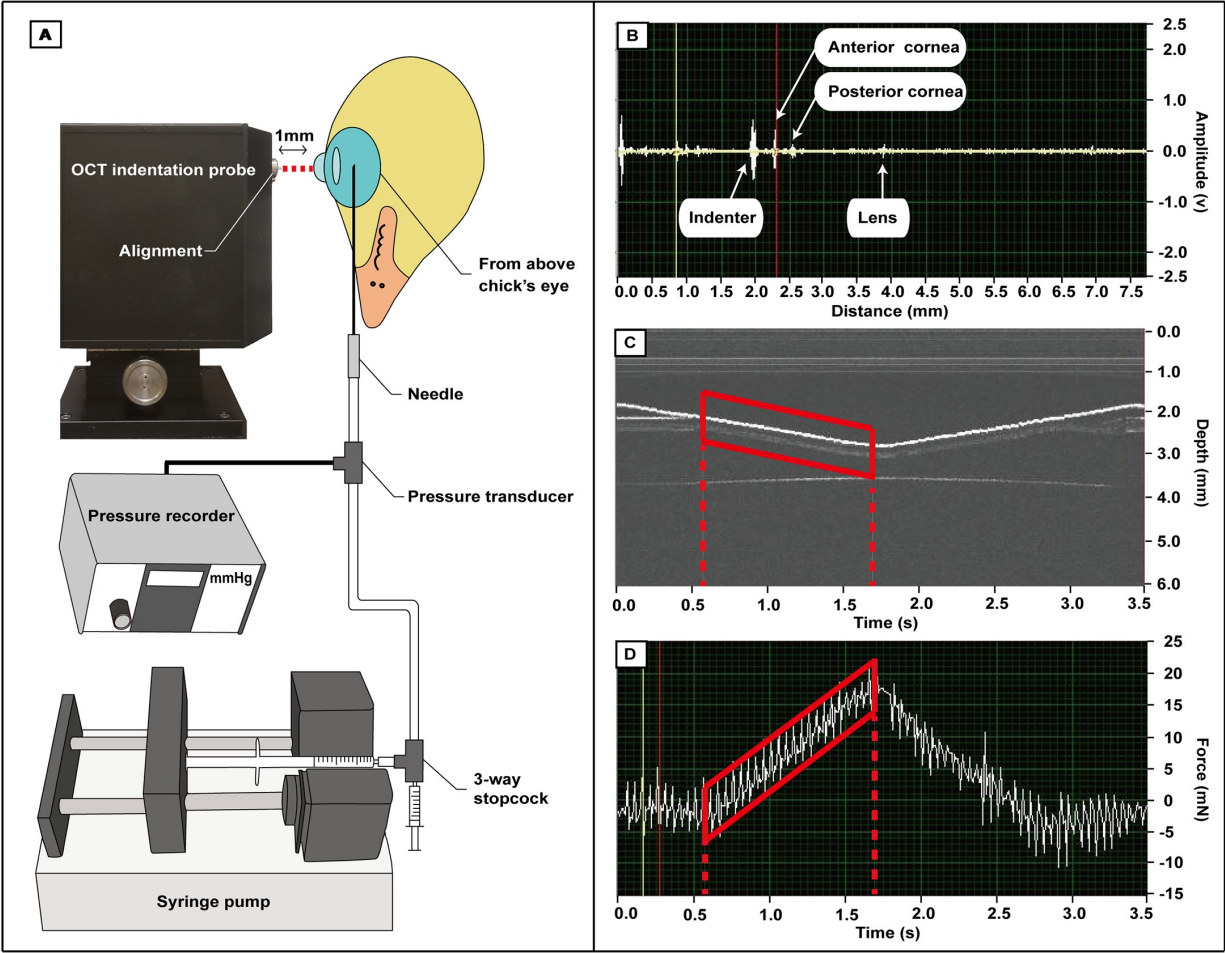
Overview of OCT-indentation probe system. (A) Schematic diagram of the set-up for OCT-indentation probe with an IOP controller. The central cornea of the chick was aligned with a visible light source emitted from the OCT-indentation probe (red dashed line). Before measurements, a digitally-controlled syringe pump with a pressure recorder was connected with the eye through a needle, to hold the IOP at one of three levels. (B) To ensure axial alignment of the indentation probe with the eye, measurements started only after the operator obtained the maximal signals from the three anterior ocular surfaces (anterior cornea, posterior cornea, and anterior lens). (C & D) After each 1-mm indentation was completed (red outlines), the deformation depth (C) and corresponding force (D) over time were cross-correlated to calculate the corneal tangent modulus (TM) and corneal stiffness (CS). The oscillations in (D) were due to motor vibration, and were removed before further data analyses (see also S2 Fig).

Applying the principle of an ultrasound-indentation system[28], we developed and validated (see below) a customized optical-coherence-tomography-indentation (OCT-indentation) system, and used it to measure *in-situ* corneal tangent modulus (TM) and corneal stiffness (CS). The OCT-indentation system consisted of a fiber-optic based time-domain OCT, an indenter of 1 mm diameter, a CCD camera, and data acquisition modules (see S1 Fig for the dimensions of the system). The infrared beam was generated by a 1310 nm super-luminescent diode (SLD) light source (Dense-Light, DL-CS3055 A, Singapore) with an output power of 5 mW and a 3 dB bandwidth of 50 nm. To aid in alignment with the central cornea, a visible light source providing red light (Fig 1A) and the CCD camera were coupled into the system. The scanning depth was set at approximately 8 mm, with a fast-scanning delay line. Light scattered and reflected from the anterior ocular components (Fig 1B) was detected using the OCT A-scan mode and transformed into digital images through a data-acquisition module. The maximum indentation depth was set to 1 mm, with a speed of 0.57 mm/s (vs. 0.83mm/s of the strain rate of strip test). The indentation depth and the corresponding force (shown as red boxes in Fig 1C and 1D), recorded by a force sensor (Model JLBS-M2-10N, Bengbu Sensor System Engineering Co. Ltd. China), were displayed in real time during measurements. A custom-written algorithm (Labview, version 12, National Instrument, U.S.) was developed to control the OCT indentation system and record data. For data analysis, a MATLAB algorithm (MATLAB R2007b, Version 7.5.0, The MathWorks, U.S.) with a cross-correlation method was used to track corneal displacement (units: mm) under the corresponding indentation force. The corneal stiffness coefficient (units: mN/mm) was then derived from the regression line of indentation force vs. corneal displacement. The corneal tangent modulus (units: MPa) was calculated by taking into account the individual corneal radius of curvature and thickness, collected from VKS and A-scan ultrasonography, respectively[28–30] (see S2 and S3 Figs). Corneal tangent modulus (TM) describes the tangent modulus of elasticity (E) at a given IOP (instantaneous slope of the stress-strain curve at a specific stress or strain), taking into account the contributions of corneal thickness and corneal radius of curvature. The equation used to derive TM was adopted from previous studies [28, 29, 34]:

$${E|}_{IOP}=\frac{a(R_{c}-t∕2)\sqrt{1-v^{2}}}{t^{2}}{\frac{dF}{d\delta }|}_{IOP}$$

where *Rc* is the corneal radius of curvature, *t* is the corneal thickness, *v* is the Poisson’s ratio (0.45) [28], *dF* is the differential force, *dδ* is the displacement interval, and *a* is a geometrical constant derived from *μ*:

$$\mu =r_{0}{\left[\frac{12(1-v^{2})}{{\left(R_{c}-t∕2\right)}^{2}t^{2}}\right]}^{1∕4}$$

where *r*_*0*_ is the radius of the full-contact area between the flat-surface indentation probe and the cornea. Thus, *a* is determined by interpolating the values from the relationship between *a* and *μ* [34].

### Validation and repeatability of OCT-indentation probe

To validate the OCT-indentation system, we first determined its accuracy in measuring tangent modulus of seven silicone corneal phantoms made with a range of Young’s modulus (0.05 to 0.64 MPa) that covered the TM values of chicks (0.12 to 0.52 MPa) as revealed in a pilot experiment. The mean TM values (average of three measurements) of the seven corneal phantoms were then compared with the tensile modulus[28] measured with an extensometer (Electro Force 3600, TA Instruments, U.S.) for the corresponding corneal strips. Note that tangent modulus and tensile modulus are two distinctly different indices, derived by different methods and formulae; viz.: while tangent modulus reflects the stress-strain relationship along the deformation depth and surface, tensile modulus is derived by biaxial stress-strain relationship. The tensile modulus measurement was chosen as an external validity test, because it is known to most investigators. The corneal phantoms and strips were made by 7 sets of RT2 silicone[35] (E600~635A/B, Hong Ye Jie Technology, China). All corneal phantoms were designed to mimic the normal chick cornea’s central thickness (200 μm), radius of curvature (3 mm), and white-to-white diameter (5 mm). Each corneal phantom was mounted on an artificial anterior chamber, using a previously established set-up[28], and two sets of three OCT-indentation measurements were collected at 5 mmHg IOP. The silicone strip was first fixed by two jaws on an extensometer, and then a 20 mN pre-stress was applied to the strip with an initial length of 8mm, followed by an elongation of 6mm with a velocity of 50 mm/min. Because of the viscoelastic property of the silicone strip, a regression analysis between stress and strain was performed, and the strain from 25% to 35% on the linear slope was selected for the calculation of tensile modulus. The tangent modulus and tensile modulus, collected respectively by the OCT-indentation system and extensometer, were analyzed by a regression analysis.

The reliability and repeatability of the OCT-indentation system for measuring the tangent modulus were tested on three silicone corneal phantoms (Tensile modulus; A = 0.053 MPa, B = 0.266 MPa, C = 0.507 MPa). Similar settings as described above were employed, and two sets of three measurements were made for each of the four IOP levels (0, 5, 15, 25 mmHg) that cover the chicks’ physiological IOP range[36] (12 to 22 mmHg). Intra-class correlation coefficients (ICC) were assessed.

### Measurement of corneal biomechanical properties in chicks’ eyes

After the completion of ocular biometric measurements, the chicks were euthanized by carbon dioxide asphyxiation, to prevent the reflexive response of the nictitating membrane from interfering with the movement of the probe during indentation. While the head was maintained in an erect posture on an adjustable platform, the eyelids were held apart gently with a speculum. A computer-programmed syringe pump (NE-1000, New Era Pump, U.S.) was used to control IOP during the indentation process. This pump was connected with a 1mL syringe filled with 0.9% saline, with its infusion rate set as 0.2 mL/hr. The apparatus was handled with special care to remove any trapped air bubbles, which would degrade the accuracy of IOP increments. To measure and monitor the pressure in the eye, a 30G needle (BD Precise Glide, U.S.) was used to cannulate the eye through the superior sclera, to a depth of approximately 5 mm (around the middle of the vitreous chamber). This set-up allowed the IOP to be maintained at a pressure of up to 50 mmHg without leakage of aqueous humor. The needle was connected with a pressure transducer (BP transducer, Harvard Apparatus, U.S.) and the syringe pump through a 3-way stopcock. The hydrostatic manometer was calibrated by recording the heights of the reservoir when the pressure transducer read 0, 10, 20, 30, 40, and 50 mmHg; pressure was calculated using the equation: p = ρ×g×h; where p = pressure; ρ = density of 0.9% saline (1.0046); g = acceleration of gravity (9.81 m/s^2^; and h = height (m). When measuring the corneal biomechanical properties, the IOP levels were controlled by converting the pressure transducer’s readings–using the formula (y = 0.991x+0.470, r^2^ = 0.999), generated by a linear regression fit (S4A Fig)–to the IOPs measured by the two methods. Bland-Altman analysis (S4B Fig) showed good agreement between IOP measurements by the pressure transducer and the hydrostatic manometer (mean difference = −0.246 mmHg, 95% limits of agreement = −1.331 to 0.841 mmHg). To determine the effects of IOP on corneal biomechanical measurements, three IOP levels (5, 15, and 25 mmHg) were chosen to cover the normal physiological range of IOP (12 to 22 mmHg) in alert chicks[36]. The indentation probe was controlled by a high-precision linear stage (S1 Fig), by which it was moved towards the corneal surface and set at about 0.2~0.3 mm in front of corneal apex (Fig 1B). The CCD camera was used to align the primary location of the probe with the central cornea, using a visible light source. Afterwards, three sets of measurements were collected, when maximal signals from the anterior cornea, posterior cornea, and crystalline lens were clearly identified from the real-time OCT images (Fig 1B). The sequence for measuring corneal biomechanical properties in the two eyes was randomized, and the measurements for each eye were completed within 10 minutes.

### Statistical analyses

All statistical analyses were conducted using either IBM SPSS (version 21.0.0, IBM, U.S.) or GraphPad Prism (Version 6.01, GraphPad Software, U.S.). Normality of distribution of variables was first verified by the Shapiro-Wilk test. As the data for refraction were normally distributed, paired *t*-tests were used to test the differences in refractive status between the treated (or right) and contralateral (or left) eyes. The comparison between right and left eyes in the normal control group was tested by Mann Whitney U-test, because of the small sample size (n = 4). A mixed two-way ANOVA was performed when both normality by Shapiro-Wilk test and homogeneity of variance by Levene’s test were not violated. Depending on the result of Mauchly’s test, either Greenhouse-Geisser (if ε>0.75) or Huynh-Feldt (if ε<0.75) corrections were applied when sphericity of variance was violated. To test the main treatment effects of form deprivation and IOP levels on corneal biomechanics, *post-hoc* Bonferroni tests were used. Independent *t*-tests were conducted to test the intergroup differences in corneal biomechanics. Correlations between IOP and corneal biomechanics at different IOP levels were tested using Pearson’s correlation analysis. Multiple regression analysis was performed to determine the contribution of ocular biometric parameters to corneal biomechanics. To prevent multi-collinearity, the minimum cutoffs for tolerance and variance inflation factor (VIF) were set as 0.1 and 5.0 respectively. Dependent variables showing a non-linear relationship with independent variables were excluded. The significance level for all tests was set at 5%.

## Results

The OCT-indentation system showed high external validity when comparing the tangent modulus (TM) measurements of this system with the tensile modulus measured by an extensometer (S5A Fig, y = 2.078x–0.0691, r^2^ = 0.96, p<0.001). The system also showed low intra-session variability (mean coefficient of variance (CV) = 8.53%) and good inter-session repeatability from two sets of three consecutive TM measurements performed on seven corneal phantoms (intraclass correlation coefficient (ICC) = 0.992; 95% confidence intervals (CI) = 0.982 to 0.997, p<0.001; see also S5B Fig for a Bland-Altman plot). When applying the system to measure TM of three corneal phantoms of different tensile modulus (0.053, 0.266, and 0.507 MPa) at four IOP levels (0, 5, 15, and 25 mmHg), the system also showed low intra-session variability (mean CV = 4.98%) and a high degree of inter-session repeatability (ICC = 0.994, 95% CI = 0.988 to 0.997, p<0.001; see also S6B Fig for a Bland-Altman plot) from two sets of three TM measurements collected at the four IOPs.

Table 1 summarizes the ocular biometric parameters (SE: Spherical Equivalent; CRC: Corneal Radius of Curvature; CCT: Central Corneal Thickness; ACD: Anterior Chamber Depth; LT: Lens Thickness; VCD: Vitreous Chamber Depth; RT: Retinal Thickness; CT: Choroidal Thickness; ST: Scleral Thickness) and corneal biomechanical properties (TM, Tangent Modulus and CS, Corneal Stiffness coefficient) measured at P12 (post-hatching day 12) in both eyes of chicks in the FD-treated and age-matched normal groups. There was no evidence of any significant difference in ocular parameters and corneal biomechanical properties (TM and CS), between the fellow untreated (left) eyes of the FD-treated group and the right and left eyes of the normal group (Mann-Whitney U-tests, all p>0.05). Furthermore, the effect sizes (G*Power, version 3.1.9.3, Universität Düsseldorf, Germany) for comparing TM of the right and left eyes of normal birds (n = 4) were all <0.29 at three IOPs–indicating low variability and negligible interocular differences, even with a small sample size. One week of form deprivation induced significantly higher myopia, and a steeper and thinner cornea, in treated eyes than in the fellow untreated eyes (Table 1, paired-*t*-test, all p<0.05).

**Table 1:** Summary of ocular biometric data and corneal biomechanical properties.

		FD-treatment Group (n = 12)			Age-matched Normal Group (n = 4)		
Parameters	Unit	RE (Treated)	LE (Untreated)	p	RE	LE	p
SE	D	-26.75±10.16	-0.34±0.84	<0.001	0.03±0.72	0.37±0.89	0.886
CRC	mm	3.07±0.10	3.19±0.09	0.001	3.17±0.07	3.19±0.06	1.000
CCT	μm	185.0±13.2	192.7±8.8	0.032	194.9±6.7	195.8±6.4	0.886
ACD	μm	1412.9±150.6	1249.1±51.0	0.004	1228.5±138.4	1323.5±173.8	0.486
LT	μm	2224.3±164.8	2074.8±86.7	0.008	2198.5±236.8	2073.4±147.3	0.686
VCD	μm	5968.5±329.4	5149±165.5	<0.001	5232.7±137.2	5225.4±192.5	0.886
RT	μm	205.3±16.9	230.6±18.6	0.020	210.4±12.5	191.5±62.8	0.686
CT	μm	167.5±44.3	223.7±32.8	0.010	233.1±39.4	263.6±17.0	0.343
ST	μm	107.8±26.5	111.9±17.7	0.661	114.9±32.9	124.1±42.7	0.886
TM @ IOP 5	MPa	0.12±0.013	0.15±0.014	<0.001	0.14±0.007	0.15±0.007	0.343
TM @ IOP 15	MPa	0.28±0.048	0.35±0.045	0.002	0.32±0.026	0.32±0.024	1.000
TM @ IOP 25	MPa	0.42±0.061	0.52±0.071	0.001	0.49±0.061	0.50±0.051	0.886
CS @ IOP 5	mN/mm	10.52±0.98	12.28±1.47	0.001	12.26±0.47	12.43±1.14	0.886
CS @ IOP 15	mN/mm	24.02±3.20	28.56±3.37	0.011	27.13±2.83	26.67±2.79	1.000
CS @ IOP 25	mN/mm	35.99±3.37	41.90±4.33	0.002	40.90±5.66	41.72±6.21	0.886

SE = spherical equivalent; CRC = corneal radius of curvature; CCT = central corneal thickness; ACD = anterior chamber depth; LT = lens thickness; VCD = vitreous chamber depth; RT = retinal thickness; CT = choroidal thickness; ST = scleral thickness; TM = tangent modulus; CS = corneal stiffness coefficient. Data are mean ± SD, paired-*t*-tests in the treated group and Mann-Whitney U-tests in the normal group.

**Fig 2:**
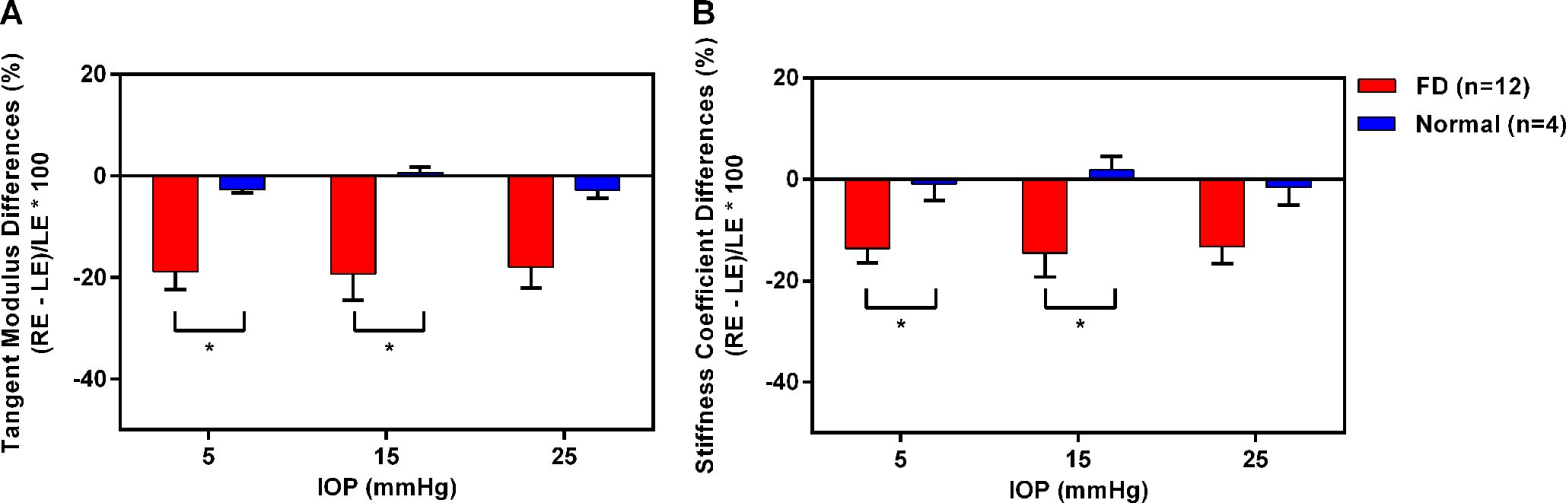
Effects of form-deprivation (FD)-induced high myopia, on corneal biomechanical properties. Significant interocular differences (100%*(RE [treated eye]–LE [fellow eye])/LE [fellow eye]) in (A) TM and (B) CS were found at 5 and 15 mmHg between the treated and normal groups. Mann-Whitney U-tests, *p<0.05. Bars represent mean ± SEM.

There were no significant differences in TM or CS, between the right and left eyes of chicks in the age-matched normal group, at any of the three IOP levels; in contrast, there were significant reductions in both TM and CS in FD-treated eyes compared to fellow untreated eyes, at all three IOP levels (mixed two-way ANOVAs, all p<0.01, Table 1). However, TM and CS were found to increase as the IOP increased, in both normal and treatment groups (mixed two-way ANOVAs, all p<0.001). Fig 2 shows the percentage difference in TM and CS between the treated and control eyes [100%*(treated eye–fellow eye)/fellow eye] in treated versus normal groups. TM and CS of treated eyes were smaller than those of normal control eyes at all three IOP levels (Table 1), with statistically significant differences at 5 and 15 mmHg (Mann-Whitney U-tests, all p<0.05) but not at 25 mmHg (Mann-Whitney U-test, p>0.05). In terms of percentage of eyes showing interocular differences in TM, eleven (92%) treated eyes showed a reduction in TM at 5 mmHg (range = −4.27% to −36.56%) and 15 mmHg (range = −2.34% to −41.44%), while ten (83%) treated eyes had lower TM at 25 mmHg (range = −4.86% to −36.81%).

**Fig 3:**
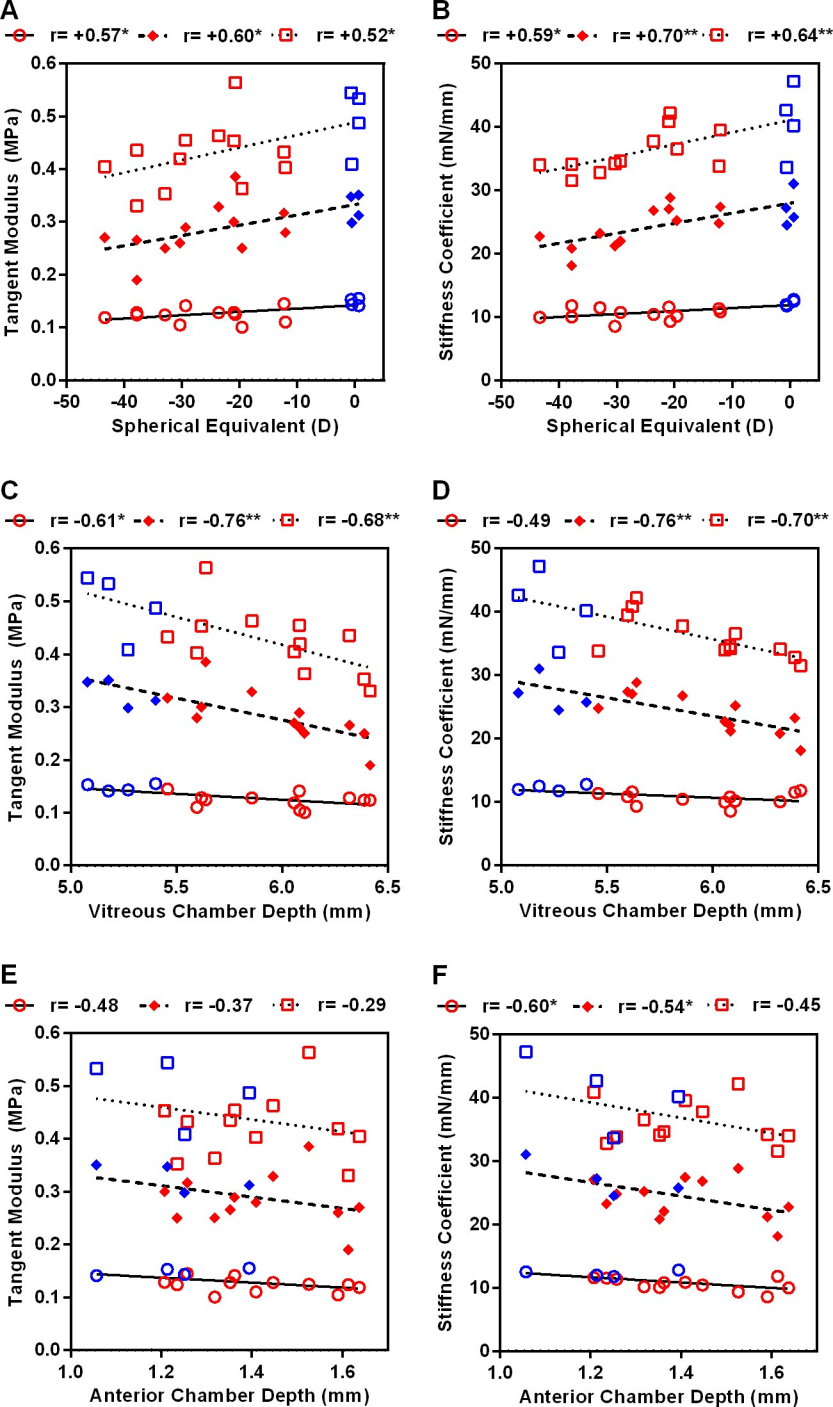
Pearson correlations between biometric parameters (spherical-equivalent refractive errors, vitreous chamber depth, anterior chamber depth) and TM and CS. Red and blue symbols represent treated and normal chicks respectively. ○: IOP 5 mmHg, ◇: IOP 15 mmHg, □: IOP 25 mmHg. *p<0.05, **p<0.01 (for correlation analyses).

Pearson correlation analyses were conducted to determine the relationship between ocular biometric parameters and corneal biomechanical properties at the different IOP levels (Fig 3). SE was moderately correlated with TM (all r>+0.52, p<0.05) and CS (all r>+0.59, p<0.05) at all IOPs, and even higher correlations were found between VCD and corneal biomechanical properties at most IOPs (TM at all IOPs: r>−0.61, p<0.05; CS at 15 and 25 mmHg: r>−0.70, p<0.01). Lastly, ACD showed moderate correlations with CS at 5 and 15 mmHg (r>−0.54, p<0.05) but was not correlated with TM (r>−0.28, p≥0.06). CRC, CCT, ST did not show any significant correlations with TM or CS.

To evaluate which ocular biometric parameters (excepting AL, because it was the sum of individual axial dimensions) play a major role in corneal biomechanics, multiple linear regression analyses were performed. Variables showing non-linearity (by using scatterplot) with corneal biomechanics, or multicollinearity with VCD, were excluded from the analyses; this left four parameters (CRC, CCT, ACD, and VCD) for further analyses. Our results (S1 and S2 Tables) showed that VCD was the only variable strongly associated with TM and with CS; this was the case under all IOP levels except 5 mmHg IOP, at which none of the tested variables showed significant associations.

## Discussion

This study showed that, in chicks: 1) the novel OCT-indentation probe provided reliable and repeatable *in-situ* corneal biomechanical measurements; 2) form deprivation produced significant reductions in corneal tangent modulus and stiffness when IOP levels were controlled at physiological levels; and 3) the two corneal biomechanical indices were significantly correlated with elongation of the vitreous chamber in highly myopic eyes.

A high degree of interest in corneal biomechanics has driven the development of multiple measuring devices using various approaches. Devices for measuring corneal biomechanical properties have evolved from conventional stress-strain measuring instruments (i.e., uniaxial tensile test[37]) and inflation tests[38] to commercially available air-puff systems (i.e., Ocular Response Analyzer, ORA; Reichert, Depew, New York[39]; and Corneal Visualization Scheimpflug imaging, Corvis ST; Oculus, Wetzlar, Germany[40]); more recent devices, still under development, include optical coherence elastography (OCE)[41], Brillouin microscopy[42], ultrasound indentation[28], and corneal indentation[29, 43]. Although the uniaxial tensile test is the gold standard in mechanical engineering, measuring corneal biomechanics using this technique is difficult, because the cornea is anisotropic, highly curved, and hydrated; furthermore, measurement along a single axis may not represent corneal biomechanics as a whole, and stretching the cornea during the measurement may disrupt the distribution of its collagen fibrils[44]. On the other hand, while the inflation test has the advantage of maintaining the cornea in physiological condition, the absence of adjacent tissues in the *in-vitro* testing condition may limit extrapolation of these measurements to the *in-vivo* condition[44]. By contrast, the air-puff systems (ORA and Corvis) measure in-vivo corneal biomechanical properties and provide multiple useful clinical parameters. Nevertheless, the parameter termed “hysteresis” (provided by ORA) is determined by multiple factors (e.g., corneal thickness, eye size), and Corvis’s Scheimpflug imaging technique may be limited by optical distortions, requiring comprehensive corrections to derive useful corneal biomechanical parameters from the raw data[45, 46]. Emerging non-contact/non-invasive devices, including OCE[41] and Brillouin microscopy[42], have extended the characterization of corneal biomechanics into mapping the elasticity distribution, but this approach awaits validation of safety and effectiveness prior to its clinical application. Another new device, using corneal mechanical indentation[29, 43], calculates the corneal biomechanics from the movement of the indenter, and thus may be confounded by any eyeball movement during measurement[28]. This inherent limitation of the corneal indentation technique can be overcome by using a newly developed ultrasound indentation technique, by means of which the tissue thickness and indentation deformation can be derived from an ultrasound signal reflected from an internal tissue interface[28].

Using an OCT-indentation device, created by adopting the operating principle of our recently-developed ultrasound indentation probe[28], we found significant reductions in both TM and CS in highly myopic eyes under all three IOP levels tested (Table 1). The method and approach we used are novel in several respects. *First*, because the cornea is the anterior-most ocular tissue, contributing to both optical quality and mechanical stability of the eye, its roles in ocular rigidity[47] and stress-strain behavior[48] have been studied widely. However, because of the cornea’s viscoelastic properties, previous biomechanical indices–derived without integrating this non-linear stress-strain characteristic (e.g., the corneal hysteresis (CH) and corneal resistance factor (CRF) generated by the Ocular Response Analyzer)–may not be sensitive enough to detect subtle structural changes in the cornea. In this study, we measured the corneal tangent modulus–Young’s modulus, derived by integrating the data within the linear portion of the stress-strain curve[28–30]–to investigate corneal biomechanical changes in an animal model widely used for studying refractive development. The biomechanical indices that we measured showed internal and external validity. *Second*, we measured both corneal tangent modulus and corneal stiffness coefficient, instead of relying only on measurements that do not take into account the corneal curvature and thickness[48]. Nevertheless, the facts that CS was only weakly correlated with corneal curvature (at all IOPs; r<–0.08, p>0.77) and thickness (at all IOPs; r<+0.12, p>0.66), and that CS was highly correlated with TM (at all IOPs; r>+0.71, p<0.01), suggest that CS can substitute for TM as a valuable metric for representing the corneal biomechanical properties in chicks. *Third*, the corneal biomechanical measurements were performed at three IOP levels covering the normal physiological range in chicks. IOP has been identified as one of the key factors influencing corneal biomechanical properties[49], and an elevated IOP has been reported in some myopic eyes[50, 51]. As revealed in Table 1 and Fig 3, the TM measurements using our OCT-indentation probe were sensitive to both the IOP level and the degree of myopia; thus, performing the measurements without controlling for IOP might have masked a potential impact of myopia on corneal biomechanics. *Fourth*, because conventional methods of assessing biomechanical properties in isolated tissue samples (*in vitro*) may cause measurement artifacts, we measured corneal biomechanical properties *in situ*. Lengthy preparation steps, as required for extensometry and inflation testing, increase the risk of structural disruption[44] and dehydration[49] of samples, which could adversely affect their biomechanical properties. In our study, the cornea was exposed to the air only during the 10-minute measurement interval, while the fellow eye was protected from dessication by the closed eyelid. Furthermore, the sequence of measurements (treated eye vs. control eye) was randomized. Consequently, our *in situ* OCT-indentation measurement should provide an assessment of the tissue’s biomechanical status under conditions very close to those of the normal cornea *in vivo*.

Our two reduced corneal biomechanical indices were associated with myopia and posterior segment depth (Fig 3). Ten of the twelve treated eyes showed reductions in corneal tangent modulus relative to that of the fellow eyes, at all three IOP levels, with the differences varying from 2% to 41%. These results–together with the growing evidence of a softer cornea in myopic eyes using different methods and animal models[13]^,^[52]–stress the importance of understanding the relationship between corneal biomechanical properties and myopia development. In this study, while the highly myopic eyes developed significant corneal thinning and steepening, along with deepening of the ACD and VCD (Table 1), VCD stood out as the key biometric parameter associated with TM and CS (Fig 3 and S1 & S2 Tables). Corneal thinning[17, 18] and steepening[14, 15] have been reported in human myopes, in some but not all studies[19–21]. In animal models of myopia, corneal steepening was found in myopic chicks[53, 54], macaque monkeys[55] and guinea pigs[56], but not in tree shrews[8, 10]. As far as we know, corneal thinning has not yet been reported in any animal models of myopia. Despite the fact that the anatomical structures of the chick and human eyes differ in many respects[31, 57], their corneas share many features, such as similar layer structure and grossly similar extracellular matrix[58]. Therefore, chicks could serve as a useful model for studying biomechanical changes of the cornea during myopia development. In further studies, it would be of interest to examine how corneal biomechanical properties alter in eyes developing myopia, and to determine whether other treatment paradigms–such as lens-induced defocus–lead to comparable biomechanical changes to the cornea.

How might corneal biomechanical properties involve in form-deprivation myopia (FDM) development? It should be noted that the biometric and biomechanical changes of the myopic cornea in this study resemble those reported in the sclera of myopes in previous studies. Specifically, in tree shrews, FDM has been shown to reduce scleral thickness at the posterior pole[11] and increase the creep rate of the sclera at both the posterior pole and equatorial region[11, 12]. FDM in chicks also increased the creep rate of the posterior and equatorial sclera, but it had no significant effects on scleral thickness or secant elastic modulus[11]–probably in part because the chick sclera includes an inner cartilaginous layer, in addition to an outer fibrous layer that is homologous to the sclera of tree shrews and other mammals, and in part because of differential molecular changes in these tissue layers during myopia development[9]^,^[59]. Because changes in creep rate in tree shrew were significantly associated with both vitreous chamber elongation and myopia severity, but not with changes in scleral thickness, it has been postulated that the axial elongation during myopia progression is regulated through the extracellular molecular changes[8, 10–12] that might alter the creep rates in mammalian sclera[11, 12]. In chicks, given the results in tree shrews[11, 12] and the lack of significant change in scleral thickness (Table 1, mean difference between treated and fellow untreated eye (mean ± SD) = ‒4.02 ± 30.89 μm; *t*(11) = ‒0.45, p = 0.66), it is less likely that a thinning-dependent process (or thinning *per se*) causes a significant reduction in scleral biomechanical properties. Nevertheless, the thinning and deformation of the cornea (Table 1) in the highly myopic chick eyes, and the significant associations between the two corneal biomechanical indices and the essential structural (vitreous chamber depth) and refractive components (spherical-equivalent refractive error), indicate that corneal biomechanical properties are sensitive to myopia development in chicks. In light of the results from tree shrews, which showed a significant association between scleral biomechanics and axial elongation rate but not axial length per se[12, 60], further studies are needed to determine the relationships (e.g., time course of change) of scleral and corneal biomechanical properties, to one another, as well as to the underlying molecular mechanisms.

Although the application of our OCT-indentation system on a myopia model provides new insights into the association between individual ocular component dimensions vs. corneal biomechanics, several improvements in methodology may be considered in future experiments. *First*, at the 1 mm-depth maximal indentation, we observed that the deformed central corneal area could be as wide as approximately 2.5 mm in diameter. Because the corneal thickness and curvature change gradually from center to periphery, the biomechanical properties we measured might be affected by the variability of these biometric properties with location in the deformed area. Whether a probe of smaller diameter might provide more accurate measurements needs to be investigated; using our current device to measure the biomechanical properties of a species with a still smaller cornea–which is likely to have greater regional structural variation–would make this potential source of bias even more important. *Second*, the corneal thickness parameter used to calculate the tangent modulus was acquired by A-scan ultrasonography 1 day before the biomechanical measurement. While this delay after A-scan was designed to prevent any adverse influence of corneal hydration (due to the application of ultrasound gel) by allowing a day for complete recovery after A-scan ultrasonography, the possibility remains that corneal thickness might have changed during that interval, or might vary during the biomechanical measurement. Real-time measurement of corneal thickness may be achieved in the future, by analyzing the two peaks representing the anterior and posterior corneal surfaces in the OCT image (see Fig 1B). *Third*, holding IOP near the physiological limit in chicks might have influenced the measurements of corneal biomechanical properties. Unlike the 5 mmHg and 15 mmHg conditions, the maximal indentation under the 25 mmHg condition led to an instantaneous increase of IOP by about 2 to 3 mmHg, as recorded by the pressure transducer. Because such a sudden increment in IOP has been associated with reduced CCT[61] and decreased ACD[62], in mammalian animal models, this might be the reason why higher standard deviations of TM and CS were noted at 25 mmHg.

In conclusion, we have demonstrated significantly lower corneal tangent modulus and stiffness coefficient in the thinner, steeper cornea of highly myopic chicks.

## Supporting information

S1 Dataset Data sets used in separate tests are organized in four Excel spreadsheets. (1) Data, contains refractive components, biometric parameters, and corneal biomechanical indices for individual birds; (2) S4 Fig, contains manometer and IOP controller data for validation test; (3) S5 Fig, contains OCT-indentation system data for external validity and repeatability tests; and (4) S6 Fig, contains tangent modulus data under four IOP levels for reliability and repeatability tests.
(XLSX)

S1 Fig Different dimensional views of the OCT-indentation system. The probe is highlighted with red circles.
(TIF)

S2 Fig Raw data for a complete cycle of indentation obtained from the OCT-indentation system. Data from a highly myopic eye (red) and the fellow control eye (white) were superimposed here to illustrate the difference. The oscillations due to the motor’s vibrations (A) were smoothened (B) before further analysis.
(TIF)

S3 Fig The process of quantifying the raw data of corneal deformation over time. (A) Time-dependent changes in corneal interface due to the indentation probe, recorded by the OCT A-scan mode. The raw data were loaded into a custom-written MATLAB algorithm for TM/CS calculations. (B) A region of interest (the corneal interface) was selected. (C) Corneal biometric parameters (thickness and curvature) from individual birds were entered for the calculation of TM. (D) The initial and peak indentation points were selected. (E) Cross-correlation analysis was performed to compute corneal biomechanical properties (TM and CS).
(TIF)

S4 Fig Validation of the IOP controller. (A) Linear regression fit of IOPs measured by a hydrostatic manometer and the pressure transducer. (B) Bland-Altman plot of the IOP readings calculated from a hydrostatic manometer and those collected from the pressure transducer. The mean difference and 95% limits of agreement are represented by a dotted line and dashed lines, respectively. ULA, upper limit agreement; LLA, lower limit agreement. The symbols represent mean ± SEM.
(TIF)

S5 Fig Validation of measurements using OCT-indentation probe. (A) External validation by examining the linear regression between the tangent modulus of the corneal phantom (by the probe) and the tensile modulus of the silicone strips from corresponding corneal phantoms (by extensometer). (B) Inter-session repeatability of the tangent-modulus measurement, by Bland-Altman plot. The mean difference and 95% limits of agreement are represented by a dotted line and dashed lines, respectively. ULA, upper limit agreement; LLA, lower limit agreement.
(TIF)

S6 Fig Reliability and repeatability of tangent modulus measurements performed on 3 corneal phantoms at 4 IOP levels using OCT-indentation probe. (A) Changes in tangent modulus of three different corneal phantoms (A = 0.053 MPa, B = 0.266 MPa, C = 0.507 MPa) under four IOP levels. (B) Bland-Altman plot of the two sets of repeated measurements of tangent-modulus under four IOP levels. The mean difference and 95% limits of agreement are represented by a dotted line and dashed lines, respectively. ULA, upper limit agreement; LLA, lower limit agreement. The symbols represent mean ± SEM.
(TIF)

S1 Table Results of the multiple regression analysis for corneal tangent modulus (independent variable) at different IOPs. (DOCX)

S2 Table Results of the multiple regression analysis for corneal stiffness coefficient (independent variable) at different IOPs. (DOCX)
