# Supplementary figures and images for: High myopia induced by form deprivation is associated with altered corneal biomechanical properties in chicks
Source: PLoS One. 2018 Nov 12;13(11):e0207189. doi: 10.1371/journal.pone.0207189 (PMC6231665; doi:10.1371/journal.pone.0207189)

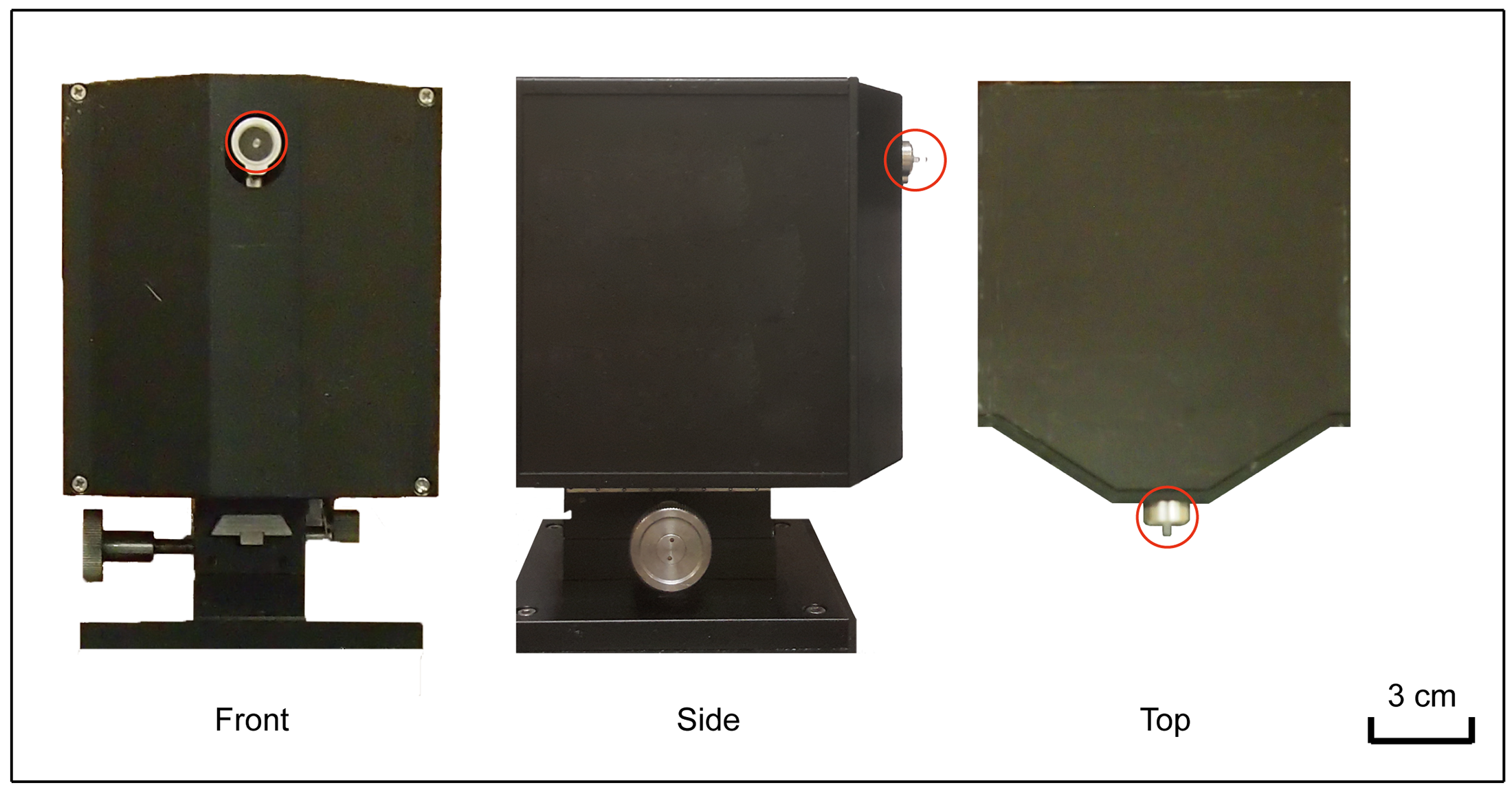

Supplement: S1 Fig — The probe is highlighted with red circles. (TIF) [file pone.0207189.s002.tif]

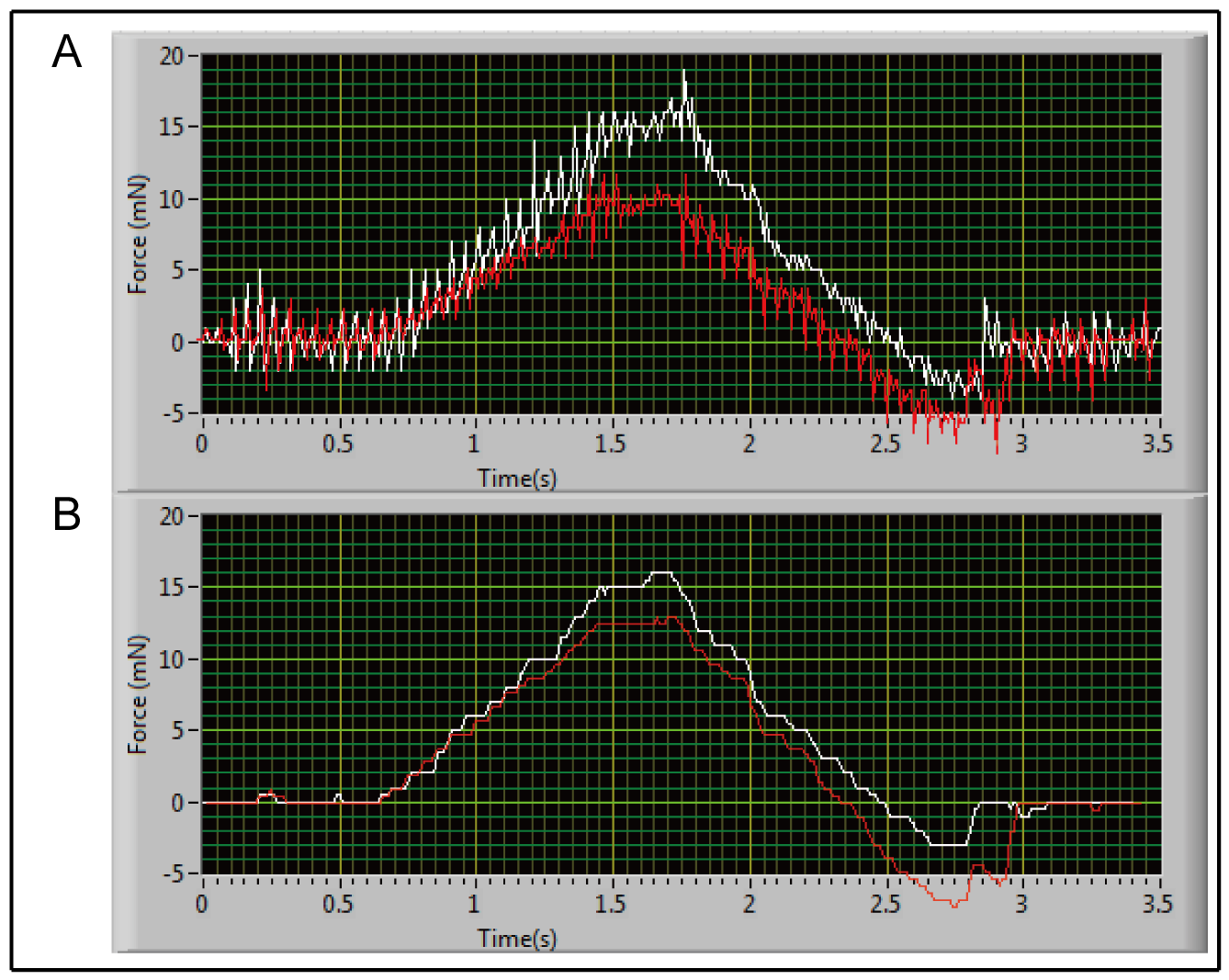

Supplement: S2 Fig — Data from a highly myopic eye (red) and the fellow control eye (white) were superimposed here to illustrate the difference. The oscillations due to the motor’s vibrations (A) were smoothened (B) before further analysis. (TIF) [file pone.0207189.s003.tif]

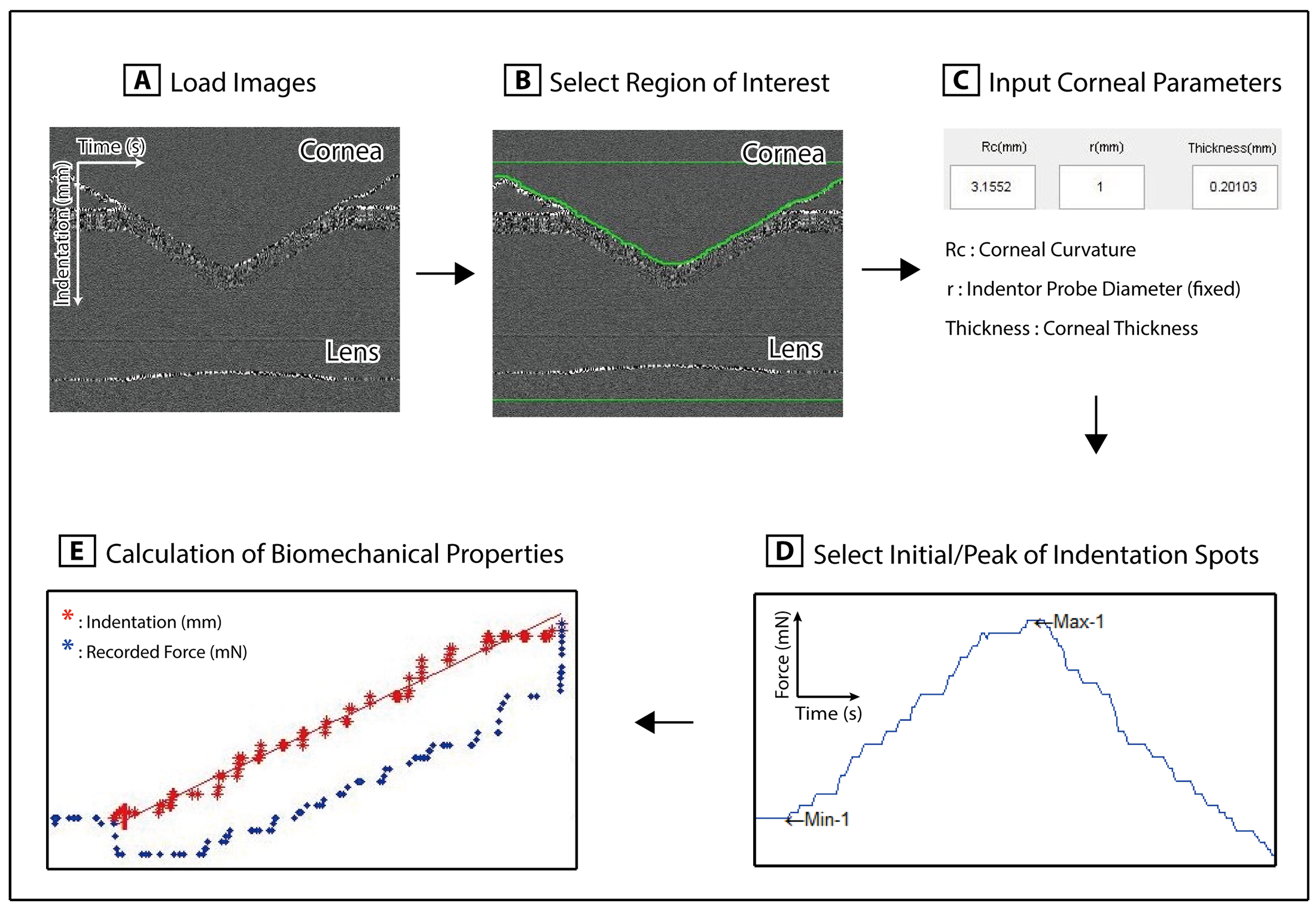

Supplement: S3 Fig — (A) Time-dependent changes in corneal interface due to the indentation probe, recorded by the OCT A-scan mode. The raw data were loaded into a custom-written MATLAB algorithm for TM/CS calculations. (B) A region of interest (the corneal interface) was selected. (C) Corneal biometric parameters (thickness and curvature) from individual birds were entered for the calculation of TM. (D) The initial and peak indentation points were selected. (E) Cross-correlation analysis was performed to compute corneal biomechanical properties (TM and CS). (TIF) [file pone.0207189.s004.tif]
