# Supplementary material for: High myopia induced by form deprivation is associated with altered corneal biomechanical properties in chicks
Source: PLoS One. 2018 Nov 12;13(11):e0207189. doi: 10.1371/journal.pone.0207189 (PMC6231665; doi:10.1371/journal.pone.0207189)
Supplement: S1 Table — (DOCX) [file pone.0207189.s008.docx]

|  | Unstandardized coefficient | |  | Standardized coefficient | t | *p* | *F* | 95% CI | Adjusted R^2^ |
| --- | --- | --- | --- | --- | --- | --- | --- | --- | --- |
|  | B | SE |  | β |  |  |  |  |  |
| 5 mmHg | — | — |  | — | — | — | 4.740* | — | .499 |
| CCT | -.001 | .000 |  | -.482 | -2.188 | .051 | — | -.001 to .000 | — |
| CRC | .076 | .034 |  | .505 | 2.260 | .045* | — | .002 to .151 | — |
| ACD | -.000 | .000 |  | -.380 | -1.662 | .125 | — | .000 to .000 | — |
| VCD | -.000 | .000 |  | -.307 | -1.326 | .211 | — | .000 to .000 | — |
|  |  |  |  |  |  |  |  |  |  |
| 15 mmHg | — | — |  | — | — | — | 7.377** | — | .630 |
| CCT | -.002 | .001 |  | -.423 | -2.231 | .047* | — | -.003 to .000 | — |
| CRC | .172 | .086 |  | .385 | 2.005 | .070 | — | -.017 to .360 | — |
| ACD | -.000 | .000 |  | -.105 | -.536 | .604 | — | .000 to .000 | — |
| VCD | -.000 | .000 |  | -.632 | -3.174 | .009** | — | .000 to .000 | — |
|  |  |  |  |  |  |  |  |  |  |
| 25 mmHg | — | — |  | — | — | — | 6.244** | — | .583 |
| CCT | -.003 | .001 |  | -.518 | -2.576 | .026* | — | -.005 to .000 | — |
| CRC | .279 | .128 |  | .444 | 2.177 | .052 | — | -.003 to .561 | — |
| ACD | -.000 | .000 |  | -.071 | -.342 | .739 | — | .000 to .000 | — |
| VCD | -.000 | .000 |  | -.570 | -2.700 | .021* | — | .000 to .000 | — |

CI: confidence interval, R^2^: adjusted coefficient of determination.

CCT: central corneal thickness, CRC: corneal radius of curvature, ACD: anterior chamber depth, VCD: vitreous chamber depth. *p<0.05, **p<0.01
