# Supplementary material for: High myopia induced by form deprivation is associated with altered corneal biomechanical properties in chicks
Source: PLoS One. 2018 Nov 12;13(11):e0207189. doi: 10.1371/journal.pone.0207189 (PMC6231665; doi:10.1371/journal.pone.0207189)
Supplement: S2 Table — (DOCX) [file pone.0207189.s009.docx]

|  | Unstandardized coefficient | |  | Standardized coefficient | t | *p* | *F* | 95% CI | Adjusted R^2^ |
| --- | --- | --- | --- | --- | --- | --- | --- | --- | --- |
|  | B | SE |  | β |  |  |  |  |  |
| 5 mmHg | — | — |  | — | — | — | 2.554 | — | .293 |
| CCT | .005 | .024 |  | .059 | .223 | .827 | — | -.048 to .059 | — |
| CRC | -3.540 | 2.894 |  | -.325 | -1.224 | .247 | — | -9.909 to 2.828 | — |
| ACD | -.003 | .002 |  | -.459 | -1.691 | .119 | — | -.007 to .001 | — |
| VCD | -.001 | .001 |  | -.353 | -1.285 | .225 | — | -.003 to .001 | — |
|  |  |  |  |  |  |  |  |  |  |
| 15 mmHg | — | — |  | — | — | — | 5.290* | — | .534 |
| CCT | -.008 | .056 |  | .030 | .139 | .892 | — | -.116 to .132 | — |
| CRC | -8.135 | 6.711 |  | -.261 | -1.212 | .251 | — | -22.907 to 6.636 | — |
| ACD | -.004 | .004 |  | -.177 | -.801 | .440 | — | -.013 to .006 | — |
| VCD | -.006 | .002 |  | -.759 | -3.397 | .006** | — | -.010 to -.002 | — |
|  |  |  |  |  |  |  |  |  |  |
| 25 mmHg | — | — |  | — | — | — | 3.787* | — | .426 |
| CCT | -.024 | .083 |  | -.067 | -.284 | .782 | — | -.206 to .159 | — |
| CRC | -10.627 | 9.901 |  | -.257 | -1.073 | .306 | — | -32.419 to 11.166 | — |
| ACD | -.004 | .007 |  | -.133 | -.545 | .597 | — | -.018 to .011 | — |
| VCD | -.007 | .003 |  | -.740 | -2.985 | .012* | — | -.013 to -.002 | — |

CI: confidence interval, R^2^: adjusted coefficient of determination.

CCT: central corneal thickness, CRC: corneal radius of curvature, ACD: anterior chamber depth, VCD: vitreous chamber depth. *p<0.05, **p<0.01.
